# Towards a climate-resilient primary health care service

**DOI:** 10.4102/safp.v65i1.5749

**Published:** 2023-09-26

**Authors:** Christian L. Lokotola

**Affiliations:** 1 Department of Family and Emergency Medicine, Faculty of Medicine and Health Sciences, Stellenbosch University, Cape Town, South Africa

**Keywords:** primary health care, climate change, air pollution, family doctors, resilience

## Abstract

Climate change has been declared as the biggest threat to human health in the 21st century. Not all family doctors are aware of the threats and how to tackle them. There are three key aspects to consider: the health and social effects of climate change, the challenge of climate change to primary health care (PHC) facilities and services, and the contribution of health services to the problem of climate change. Climate change and global pollution are ecological drivers associated with significant health and social effects that are often seen in PHC services. These ecological drivers impact health and society via a number of proximate causes, such as air pollution and decreased food production. The health and social effects include malnutrition, infectious diseases, non-communicable diseases, displacement and migration, and mental health problems. Climate change-induced extreme weather events are associated with immediate loss of life and injuries, destruction of homes and livelihoods, and disruption of PHC facilities and services. For adapting to these challenges, the World Health Organization has developed an operational framework for a climate-resilient health system. The Global Green and Healthy Hospitals agenda provides practical guidance for mitigating the contribution of health services to climate change. This article uses these frameworks to suggest practical steps that family doctors can take in leading climate adaptation and mitigation within PHC.

## Introduction

We are facing an environmental crisis that has climate change and global pollution as two of the key ecological drivers.^1^ Climate change has been declared as the greatest threat to human health in the 21st century.^2^ The interaction between climate change and health can be considered through three different aspects: the health and social effects of climate change, the challenge of climate change to (primary) health care (PHC) facilities and services, and the contribution of health services to the problem of climate change.^1^ This article considers the implications of these interactions for a climate-resilient PHC practice and the role of the family doctor. Climate resilience is the capacity to anticipate, respond to, cope with and recover from the effects of climate change.^3^

## The health and social effects of climate change

**FIGURE 1:**
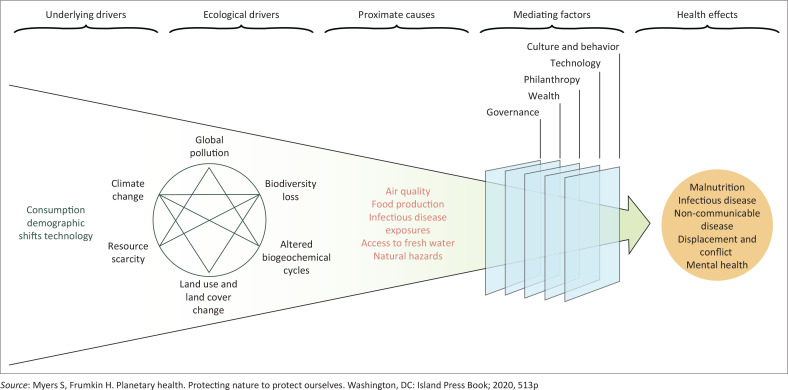
The health and social effects of the environmental crisis.

Figure 1^4^ shows how the environmental crisis impacts on health and the social determinants of health. Changes in morbidity and mortality will be seen in primary care practices, although the attribution to climate change may not always be obvious.

The health and social effects of these ecological drivers are often determined by a number of proximate causes and mediating factors. Climate change and air pollution are interlinked. In South Africa, coal-fired power stations are a major cause of greenhouse gases and air pollution.^5^ This air pollution is also a major risk factor for deaths from non-communicable diseases such as chronic obstructive pulmonary disease, diabetes, ischaemic heart disease, lower respiratory tract infections, lung cancer and strokes. Air pollution also has strong links to asthma and premature births.^6^ Warmer temperatures also convert air pollution into adverse smog, resulting in increased respiratory and cardiovascular diseases.^7^

Worsening food production is already seen in many countries as climate impacts both farmers and pastoralists.^8^ African food productivity has been reduced by 34% since 1961.^8^ Drought, heat, storms and wildfires all reduce productivity and shorten growing seasons. In addition, there are changes in the pests and diseases that attack the key crops. There is also evidence that the nutritional value of the crops may be reduced. Marine and freshwater fisheries will also be adversely affected by rising temperatures. The end result of all these changes is an increase in malnutrition and micronutrient or vitamin deficiencies.^9^

Exposure to infectious diseases is also changing. Vector-borne diseases, such as malaria, shift with changes in the vector’s habitat. For example, mosquitoes may spread to higher altitudes with warming and flooding may amplify the breeding of mosquitoes.^10^ Water-borne diseases, causing gastroenteritis, may also change because of both decreases in the quality and quantity of water as well as flooding and poor sanitation. In January 2023, South Africa saw imported cases of cholera into Gauteng from flooding in Malawi.^11^

Natural hazards include extreme temperatures as well as extreme weather events. Extreme temperatures pose a risk to people from insomnia, dehydration, heat exhaustion and heat stroke.^6^ In January 2023, extreme heat and heatwaves over 50 °C were associated with the deaths of farm workers in the Northern Cape.^12^ High temperatures have also been associated with skin problems, psychological stress, civil conflict and risks for pregnant women.^13^

In sub-Saharan African regions, frequent cyclones and massive rainfalls cause unprecedented floods and destruction. The cities of Lagos and Kinshasa have experienced frequent floods after intensive rainfalls. In March 2019, Mozambique, South Africa, Zimbabwe and Botswana were heavily impacted by Cyclone Freddy and previously by Cyclone Idai.^14^ In 2022, the city of Durban had over 400 deaths from a ‘rain bomb’, a wet microburst of excess in rainfall and thunderstorms associated with serious damage. These extreme weather events cause immediate loss of life and injuries as well as destroying homes and livelihoods.^15^

All of these health effects and proximate causes can challenge people’s coping skills and lead to mental health problems such as depression, anxiety and post-traumatic stress. These are compounded by the social effects on income, housing, family stability and the consequences of being displaced or having to migrate.^4^ It is estimated that in sub-Saharan Africa over 3.4 million people were displaced by weather-related events in 2019 and up to 40 million may migrate internally by 2050 because of global warming.^16^ Migration has additional effects on health because of people’s vulnerability (e.g., gender-based violence) and poor access to health services.^17^ Primary health care services can expect to see more migrants with unusual diseases and the associated challenges of language and cultural barriers.

The effects will be felt most by those who are more vulnerable as a result of age, gender, poverty, marginalisation or a lack of access to basic services. Some people and communities have more resources and a better ability to adapt or be resilient in the face of these challenges. The strength of PHC will itself be one of the mediating factors between climate change and health effects. Community-orientated primary care (COPC) with its focus on the whole population at risk can be a key lever in improving the preparedness and resilience of communities.^18^ For example, through addressing informal settlements that are built in flood plains or urban greening spaces that helps reduce temperatures. Primary health care needs to support and become part of COPC as our model of care and service delivery.^19^ The role of COPC underlines an opportunity to meet the demands of an evolving healthcare system based on comprehensive care, definition of health needs and determinants, and prioritisation of those needs. This implies integrating promotion, prevention and treatment, and involving the community in all processes.

## Adapting to the challenge of climate change to primary health care facilities and services

Family doctors need to be ready to collaborate in local emergency preparedness and disaster response. Several short reports and scoping reviews have underlined the difficulty that PHC facilities and services have to continue operating after extreme weather events.^20^ In Durban, some health centres are still closed a year after their ‘rain bomb’ and in Beira, Mozambique much of the PHC infrastructure was destroyed. High temperatures and water scarcity have also challenged PHC service delivery.^20^

**FIGURE 2:**
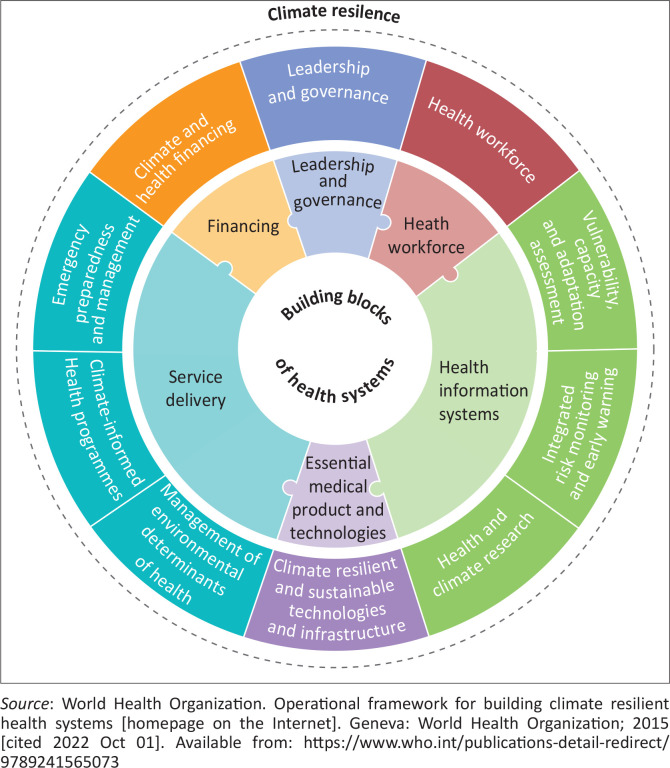
Key components of climate-resilient health services.

The World Health Organization (WHO) has developed an operational framework for climate-resilient health systems. This framework (Figure 2^3^)is built upon the usual building blocks of the health system. The building blocks are leadership and governance, service delivery, health information systems, health workforce, essential medical products and technologies, and financing.^4^ The framework helps the healthcare leaders to design a strategic plan to adapt to climate change risks and to bring about sustained improvement of climate resilient readiness healthcare system functions. It also presents key performance indicators for health system climate actions.^4^ The framework supports the healthcare system to customise their adaptation and mitigation strategies. It is a guidance plan for assessing climate-related impacts and designing possible related health and healthcare system actions around relevant indicators.

Family doctors need to think through the likely climate-related challenges and prepare for them. They need to consider if their equipment and infrastructure are appropriate to withstand the expected challenges. Most family doctors have not been trained to assess and manage temperature-related conditions. However, they can check if point of care devices works well at high temperatures. They need to control and keep temperatures down to safe limits within health facilities. Family doctors will not do the work of the electricians, but they need to check if the facility can withstand an extreme weather event and maintain its structural integrity, communications, water and energy supplies. For instance, family doctors should check and control temperature-sensitive storage and transportation of medications. They need to check if the practice can handle a ‘rain bomb’, flooding, very high temperatures or an influx of migrants. Family doctors should offer leadership within their practices and clinical teams. They can also be advocates in the broader national and sub-national dialogues to ensure that policymakers and the public are aware of the health effects. They can also lead through role-modelling environmentally sustainable behaviour and by being environmental stewards.

Family doctors need to pay attention to non-traditional health indicators that can give warning of impending health effects. A successful climate change resilient healthcare service will undoubtedly demand an increase in collecting health facility and community-level data where the healthcare facility is located for routine reports.^21^ This is often monitored by other institutions and the indicators are not connected to the health system directly. Data collection should be driven by robust, integrated health information and data management of environmental challenges to allow a rapid response. Family doctors are often under-equipped for these activities and need training. They can also work in collaboration with the sector concerned around non-traditional indicators to collect relevant information.

The various health programmes need to consider and prepare for the impact of climate change. Family doctors need to prepare themselves and their teams as essential members of the PHC workforce to be ready for the challenges. Training, mentoring and other means of education can contribute to capacity building of the health workforce. They need to think about ways to respond to food insecurity, malnutrition and the needs of migrants when accessing health services. They need to think how to manage cases of heat exhaustion or be prepared for outbreaks of diarrhoea. The World Organization of Family Doctors (WONCA) has offered a free online course on planetary health.^22^ The Southern African Association of Health Educators (SAAHE) has recently published a position statement on planetary health and environmental sustainability in African health professions education.^23^ For example, training can focus on new or unusual conditions that might be encountered or on emergency preparedness. It is also important to understand the links between the environmental crisis and local health issues in one’s community as well as the possible social effects of climate change.

A climate-resilient financing approach will anticipate the financial contingencies needed to respond to climate change. For example, in Durban, there was no funding available to replace medication lost during the ‘rain bomb’.^15^ Global financing for loss, damage and adaptation needs to be mobilised within the health sector.^24^ Table 1^20^ lists examples of how family doctors can help PHC facilities and services adapt to the challenges of climate change, matched to the components of the WHO’s operational framework for building climate-resilient health systems.^3^

**TABLE 1:** Examples of initiatives to adapt to the challenge of climate change in the primary health care facilities and services.

Components	Examples
Leadership and governance	Initiate discussion on effective responses to climate change-related health risks that affect healthcare services, and the community where the PHC facility is located Set tasks, responsibilities (to assess, to monitor and to manage), and accountability mechanisms around these responses Form a task force of representatives from various departments to help implement environmental sustainability efforts Collaborate with different stakeholders to address environmental health risks and conduct surveillance at the facility and community
Health workforce	Organise or participate in online/face-to-face training (webinar and workshop) or short courses to develop technical and professional capacity on climate change and health topics Imbed planetary health in the training of PHC providers Support individual and general efforts to acquire adequate baseline levels in climate health policy and management, research and analysis, healthcare and community health service delivery
Vulnerability, capacity and adaptation assessment	Use available tools for vulnerability assessment of the PHC facility and the community where the PHC facility is located Be aware of the most common climate change risks where the PHC facility is located Assess which population and areas are most vulnerable Define the linkage between climate risk and health effects as a baseline analysis to develop protective measures and selection of priorities Identify the weaknesses of healthcare service delivery and seek expertise to address them
Integrated risk monitoring and early warning	Use different instruments (e.g. mobile phone, community radio, risk mapping and early warning infographics) to collect qualitative and quantitative information/data about loss and damages and health effects in the facility and the community affected by climatic and local environmental conditions Always think to generate a holistic perspective of health risks with real-time information Utilise health workforce and intersectoral collaboration for an adequate response
Health and climate research	Initiate internal or external collaborative research (with interested stakeholders, for example, NGOs, universities’ health students and academics) to reduce uncertainty and to gain insight into potential solutions and capacities, guidance on priorities and to build evidence to strengthen decision-making
Climate resilient and sustainable technologies and infrastructure	Budget and invest in specific technologies that can reduce vulnerability to climate risks, both within and outside the PHC facility Ensure that the PHC facility accounts for carbon, energy, water and sanitation footprints and that the facility is prepared for future climatic and environmental risks (e.g., floods and indoor extreme temperatures) Adopt transport means that reduce the hospital’s carbon footprint
Management of environmental determinants of health	Initiate intersectoral collaboration to advocate for health-related policies in other sectors (e.g., water, agriculture, transport, housing and energy) in order to reduce health risks from the environmental determinants of health
Climate-informed health programmes	Make use of different climate resilient information (e.g., early warning about potential outbreaks, climate risk and vulnerability assessment tool) to inform the surveillance decision, facility, and community preparedness and adaptation strategies (e.g., occupational health exposure, energy-efficient cooling and heating system, and ventilation of the facility)
Emergency preparedness and management	Understand the climate and health risks to the PHC facility and population Make the emergency unit available, with robust equipment, sustainably during extreme weather events Get monthly reports from the emergency unit on the infrastructure for water supplies, drainage, waste disposal and sanitation, as well as telecommunication, energy supplies and medical transport
Climate and health financing	Prepare a budget to expend resources and to achieve climate-resilient PHC goals Seek additional investment when needed Allocate finances for adequate training of health professionals in climate-resilient PHC (trained workforce and basic infrastructure and services) to address climate risks

*Source*: Lokotola CL, Mash R, Naidoo K, Mubangizi V, Mofolo N, Schwerdtle PN. Climate change and primary health care in Africa: A scoping review. J Climate Change Health. 2023;11:100229. https://doi.org/10.1016/j.joclim.2023.100229

PHC, primary health care; NGO, non-governmental organisation.

## Mitigating the contribution of health services to climate change

South Africa has higher annual carbon dioxide emissions per capita than many European and highly developed countries (7.34 tonnes per person vs 5.15 tonnes in the United Kingdom [UK]).^25^ This is driven to a large extent by its dependence on coal as a source of energy. The global health sector also has a relatively large carbon footprint, accounting for over 4% of all carbon emissions.^26^ If the health sector were a country, it would be the fifth-largest emitter on the planet. The health sector, therefore, has a responsibility to mitigate its carbon footprint. Primary health care facilities and district hospitals have to consider how they can reduce the harm they do to the environment and by extension to the communities that they serve. Many of these initiatives are also beneficial for health, can improve resilience and can be cost-saving in the long term.

**FIGURE 3:**
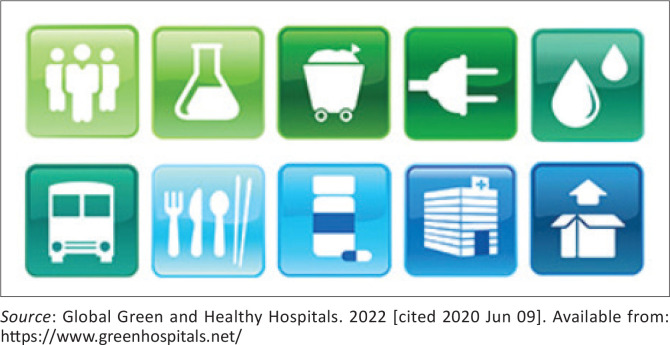
The Global Green and Healthy Hospitals agenda items: Leadership, chemicals, waste, energy, water, transport, food, pharmaceuticals, buildings and procurement.

The Global Green and Healthy Hospitals (GGHH) network has identified the 10 most important areas to consider in reducing the environmental harm of the health sector (Figure 3).^27^

Leadership from family doctors is again important in mitigation. In the public sector, some provincial governments, such as the Western Cape, have committed to becoming carbon net zero by 2050.^28^ Table 2^29^ lists examples of how family doctors could play a leading role in implementing the GGHH agenda to mitigate the environmental harm of the health sector.

**TABLE 2:** Examples of initiatives to mitigate the environmental harm of the health sector.

GGHH agenda item	Examples
Leadership	Evaluate your carbon footprint Promote behaviour change Dedicate staff resources to address environmental health issues at the facility Form a task force of representatives from various departments to help implement environmental sustainability efforts Collaborate with different stakeholders to address environmental health risks and conduct surveillance at the facility and community Provide opportunities for the education of staff and community to engage and act for environmental health and disease prevention
Chemicals	Adopt policies and ensure that chemicals undergo basic toxicity testing Replace as soon as possible products of high risk with alternatives Replace all mercury thermometers and blood pressure devices with safe, accurate and affordable alternatives Implement a facility-specific chemicals action plan
Waste	Avoid incineration of medical waste Dispose of the medication safely Reduce waste and recycle where possible Avoid single-use plastic
Energy	Install renewable energy systems such as solar energy Install more efficient appliances and systems for heating, cooling and lighting Change behaviour
Water	Implement water conservation strategies to aspire to net-zero water use in the facility Regularly analyse water quality Provide facility with potable water Develop a joint project with the community to improve and protect the water supply Consider harvesting rainwater or recycling water
Transport	Adopt transport means that reduce the hospital’s carbon footprint
Food	Reduce hospital environmental footprint while fostering healthy food Support local sustainable sources of food in the community
Pharmaceuticals	Avoid greenhouse gases from older inhaler devices Look at avoidable leakage of anaesthetic gases
Buildings	Plan new buildings with at least a 4-star green rating
Procurement	Use green cleaning products Shorten supply chains – Use local where possible

*Source*: Wyssusek K, Chan KL, Eames G, Whately Y. Greenhouse gas reduction in anaesthesia practice: A departmental environmental strategy. BMJ Open Qual. 2022;11(3):e001867. https://doi.org/10.1136/bmjoq-2022-001867

GGHH, Global Green and Healthy Hospitals.

In addition to vulnerability assessment for climate change adaptation, family doctors identify carbon footprint hotspots in their facility. The hotspots analysis can assess the 10 areas of GGHH to evaluate each department, ward and services materials used and source of carbon emission and pollution. The energy sector is the main contributor to higher carbon emissions. This brings about a strategic commitment to increase energy efficiency, reduce unnecessary usage of electricity, and understand where energy supply resilience can be improved. The theatre, procurement and waste management carry significant global warming potentials of carbon dioxide (CO_2_).^29^ Significant energy consumption is identified for inhaled anaesthetic gases such as sevoflurane, isoflurane and desflurane. One bottle of sevoflurane (250 mL) during anaesthesia is the CO_2_ equivalent of 196 km in petrol fuel car.^29^ Family physicians can outsource appropriate engineering expertise to address the clean, low-carbon and modern energy system. Family doctors need to emphasise the operational use of hybrid and renewable energy supply mostly in the carbon emission hotspot spaces.

A leadership position is required to engage the facility to join the healthcare without harming the community and learn about transitioning equitably and inclusively to net zero emission. Their leadership position creates an environmentally sustainable healthcare system that improves, maintains or restores health while minimising negative impacts on the environment. These mitigation strategies contribute to leverage opportunities to improve the benefit of the community health where the PHC facility is located.

## Conclusion

This study has explored the impact of climate change on health and the implications for PHC and family doctors. In the face of growing health and social impacts of climate change, the role of PHC is to improve the preparedness and resilience of communities where PHC facilities are located. Family doctors have to define the health needs of communities to meet the demands of climate-resilient care. They should practise intersectoral collaboration and use non-traditional health data and indicators of environmental challenges. They need to understand the components of climate-resilient PHC services in order to identify and respond to health system challenges. Family doctors can play a leading role in adapting PHC facilities and services to the challenges of climate change, as well as mitigating the considerable environmental harm of healthcare.
